# Association between Dietary Inflammatory Index and Hyperemesis Gravidarum

**DOI:** 10.3390/nu16162618

**Published:** 2024-08-08

**Authors:** Shihan Zhi, Lan Zhang, Wenjie Cheng, Yuan Jin, Zhaoqing Long, Wei Gu, Le Ma, Shunming Zhang, Jing Lin

**Affiliations:** 1 School of Public Health, Xi’an Jiaotong University Health Science Center, Xi’an 710061, China; zhishihan2000@163.com (S.Z.); zhanglan@stu.xjtu.edu.cn (L.Z.); chengwj20@163.com (W.C.); jinyuan0828@stu.xjtu.edu (Y.J.); long18090799669@163.com (Z.L.); male@mail.xjtu.edu.cn (L.M.); 2 School of Nursing, Xi’an Jiaotong University Health Science Center, Xi’an 710061, China; 232guwei@xjtu.edu.cn; 3 Key Laboratory for Disease Prevention and Control and Health Promotion of Shaanxi Province, Xi’an 710061, China

**Keywords:** dietary inflammatory index, DII, inflammation, hyperemesis gravidarum

## Abstract

(1) Background: Diet holds a pivotal position in exacerbating or ameliorating chronic inflammation, which has been implicated in the pathogenesis of hyperemesis gravidarum (HG). However, no study has explored the association between dietary inflammatory potential and HG. This study aimed to investigate the potential correlation between following a pro-inflammatory diet and the likelihood of developing HG. (2) Methods: A total of 2033 Chinese pregnant women (mean age: 31.3 ± 3.4 years) were included in this cross-sectional study from April 2021 to September 2022 as part of the China Birth Cohort Study (CBCS). Dietary inflammatory index (DII) scores with 23 food components were constructed through dietary intakes collected via a reliable 108-item semi-quantitative food frequency questionnaire. HG was defined as a pregnancy-unique quantification of emesis (PUQE) score ≥13 points, severe nausea and vomiting leading to weight loss ≥5%, or being hospitalized for treatment due to the disease. The relationship between DII and HG was conducted utilizing binary logistic regression and restricted cubic spline regression. (3) Results: Overall, 8.2% (*n* = 167) of study participants had HG. The DII scores ranged from −4.04 to 3.82. After adjusting for potential confounders, individuals with the highest tertile of DII score had a higher risk of HG (OR = 1.65, 95% CI: 1.04, 2.62, *P*_trend_ = 0.032). Such an association was stronger in those with pre-pregnancy overweight/obesity (*P*_interaction_ = 0.018). (4) Conclusions: A higher DII score, which serves as a marker for a diet promoting inflammation, is correlated with an elevated risk of developing HG. This finding suggests that dietary recommendations for HG should focus on minimizing the DII through incorporating foods abundant in anti-inflammatory components.

## 1. Introduction

Hyperemesis gravidum (HG) refers to severe and persistent nausea and vomiting in pregnancy (NVP), consisting of dehydration, electrolyte disorder, weight loss, ketosis, and even acidosis in early pregnancy that requires hospitalization [1]. The incidence of HG in pregnant women globally ranges from 0.3% to 10.8% [2]. Growing evidence has revealed a significant link between HG and an augmented risk of maternal health complications (such as anemia, eclampsia, venous thromboembolism, and gestational hypertension disorder, etc.) [3] and adverse perinatal outcomes (e.g., fetal growth restriction, low birth weight, and premature delivery), which has a long-term impact on the health of offspring [4,5]. Thus, it is crucial to pinpoint potentially alterable risk factors, such as diet, in order to prevent the development of HG.

Inflammation has been hypothesized as a potential contributor to HG. In particular, inflammation markers, such as platelet-to-lymphocyte ratio (PLR), monocyte-to-lymphocyte ratio (MLR), and neutrophil-to-lymphocyte ratio (NLR), have been associated with increased risk of HG [6,7]. Moreover, the supplementation of anti-inflammatory foods and nutrients such as ginger and vitamin B6 is effective in mitigating the symptoms of NVP [8]. This suggests that a diet with anti-inflammatory capacity may be related to the alleviation of HG clinical symptoms. In this regard, our previous study also observed a noticeable inverse association between dietary patterns rich in anti-inflammatory foods, such as fish, shrimp, eggs, and milk, and the occurrence of HG [9]. However, the reports on the potential inflammatory effects of diet and the development of HG are limited. The potential link between dietary components and HG might be modulated by inflammation [10], and researchers have utilized the dietary inflammatory index (DII) to assess the diet’s propensity to elicit an inflammatory response. DII serves as a comprehensive, literature-grounded instrument, enabling the evaluation of an individual’s dietary pro-inflammatory or anti-inflammatory potential [11]. Several studies have verified a correlation between DII and various maternal and infant complications, such as hypertension disorders of pregnancy [12], gestational diabetes mellitus (GDM) [13], congenital heart disease [14], and preterm birth [15]. However, the existence of a correlation between DII and HG remains unconfirmed.

Therefore, this study endeavors to explore the potential relationship between the DII and the risk of HG among Chinese pregnant women. The findings could provide valuable information for the effective prevention and treatment of HG during early pregnancy, which in turn may help reduce the occurrence of adverse maternal and infant outcomes.

## 2. Materials and Methods

### 2.1. Participants

This cross-sectional study, nested within the China Birth Cohort Study (CBCS), aimed to investigate the potential correlations between maternal nutrition, lifestyle habits, medication intake, and the health outcomes of both mothers and their offspring, whose profile and details have been previously reported [16,17]. Briefly, pregnant women aged 18~49 years at their first prenatal care visit were enlisted for participation in the cohort. Sociodemographic, lifestyle, and anthropometric variables were collected using a structured electronic questionnaire when the participants first entered the cohort, and obstetric information was obtained through the Hospital Information System (HIS) of the hospital. Participants in the study, which ran from April 2021 to September 2022, were recruited from the Northwest Women’s and Children’s Hospital. The inclusion criteria were as follows: (1) pregnant women who were ≥18 years old, within the gestational period of 6 to 16 weeks, who consented to participate in this study; (2) participants with the ability to complete the questionnaire in Chinese. Women with any cognitive or psychiatric illness and other medical causes of nausea and vomiting were excluded.

To calculate the required sample size, we used the formula suggested by Cross et al. [18]. We hypothesized the prevalence of HG as 10.8%, allowable error as 2%, and type I error as 0.01. The threshold for the necessary sample size for the present analysis was 1604. To mitigate potential bias stemming from missing data and lost access, the study enrolled a total of 3122 women, with 607 participants failing to complete all the questionnaires and 482 having some missing data. As a result, the final sample size consisted of 2033 women. Written informed consent was obtained from all participants, and the study was granted approval by the Ethics Committee of the Department of Medicine, Xi’an Jiaotong University (No. 2020-1263).

### 2.2. Assessment of Dietary Intakes

Dietary intake data were gathered utilizing a semi-quantitative Food Frequency Questionnaire (FFQ), tailored from a previously validated FFQ specifically designed for pregnant women residing in rural western China. The reliability and validity of this adapted FFQ ranged between 0.40 and 0.80 [19,20]. The FFQ covered 10 principal food categories, specifically cereals, vegetables and their products, beans and their products, fungi and algae, fruits, nuts, meat and eggs, dairy products and their derivatives, alcoholic beverages and drinks, and candies and baked goods, with a total of 108 food items. It also included the average monthly intake of edible oils and animal fats. A standard serving size was established for each food item included in the list. Pregnant women filled out the FFQ by recalling the average amount consumed per month, per week, or day over the past year. A food atlas (a specific amount of each food) provided in the questionnaire assisted participants in approximating the quantity of food they had consumed. The frequency of intake for each food was classified into nine levels: none or <1 serving/month, 1–3 servings/month, 1 serving/week, 2–4 servings/week, 5–6 servings/week, 1 serving/day, 2–3 servings/day, 4–5 servings/day, and ≥6 servings/day. Then, we unified the food intake frequency into serving/day. The nutrient content per 100 g of a certain food was looked up in the China Food Composition Tables Standard Edition (Book 1, 2nd edition). Finally, the daily intake of a certain nutrient can be calculated by summing up the products of the number of servings of each food consumed per day, the weight of each serving, and the content of that nutrient in 100 g of that food, and then dividing the result by 100 g.

### 2.3. Assessment of DII

The dietary information collected through the FFQ was utilized to calculate DII scores for each participant. This index was based on a comprehensive assessment of 45 distinct foods and nutrients, each of which was associated with either a pro-inflammatory or anti-inflammatory effect [11]. Given the distinctive dietary habits prevalent in Chinese culture, where foods like eugenol, saffron, and rosemary are not frequently consumed, and considering the limitations of our nutrient database which lacks information on items like pepper, trans fats, and turmeric, we calculated the DII score using 23 food parameters, as opposed to the full set of 45 food parameters; pro-inflammatory parameters included energy, carbohydrate, total fat, protein, cholesterol, vitamin B12, iron, and alcohol, and anti-inflammatory parameters included monounsaturated fatty acid (MUFA), polyunsaturated fatty acid (PUFA), fiber, vitamin B6, folic acid, niacin, riboflavin, thiamine, vitamin A, vitamin C, vitamin D, vitamin E, zinc, selenium, and magnesium. Consistent with previous studies [11], DII was calculated by converting the standardized individual food parameters intake (through mean and standard deviation of daily intake per capita worldwide) into percentile values and in turn multiplying the corresponding inflammatory effect score of each food parameter. For example, if a food parameter promoted the elevation of inflammatory markers like interleukin-1beta (IL-1β), interleukin-6 (IL-6), C-reactive protein (CRP), and tumour necrosis factor-alpha (TNF-α) or decreased the levels of anti-inflammatory cytokines, e.g., interleukin-4 (IL-4) and interleukin-10 (IL-10), it was assigned a score of “+1”. Conversely, if it had the opposite effect, it was assigned a score of “−1”. If there was no effect, it was assigned a score of “0”. Ultimately, we summed each score for the total DII scores [11,21]. A higher DII score served as an indicator of a diet with greater inflammatory potential, whereas a lower DII score signified a diet with reduced inflammatory properties. To investigate the relationship between DII scores and HG, participants were categorized into groups based on the tertile thresholds of their DII scores.

### 2.4. Assessment of HG

The pregnancy-unique quantification of vomiting (PUQE) scale was used to assess the severity of NVP symptomatology, which showed good internal consistency and reliability (0.846) and validity (0.950) [22,23]. There are three entries in the questionnaire, scoring grade 5 for daily nausea (in hours), daily retching episodes, and daily vomiting episodes. A PUQE, with a total score of 3 to 15, equal to or greater than 13 points is identified as severe NVP or HG [24].

Definitions for HG in clinical and research contexts have had a considerable level of heterogeneity. At present, persistent and severe symptoms of NVP can lead to weight loss, and a weight loss of ≥5% was an important indicator in the definition of HG [25], while the use of ketonuria to diagnose was not recommended [24]. In addition, some cases with severe symptoms required hospitalization for treatment [1,26]. Based on previous research, the diagnostic criteria for HG were as follows:

(1) PUQE questionnaire score ≥13;
(2) Inability to eat normally due to severe nausea or vomiting, seriously affecting daily living activities;
(3) Dehydration and weight loss after pregnancy exceeding 5% of pre-pregnancy weight;
(4) Seeking medical attention due to NVP and receiving medical interventions;
(5) Beginning of symptoms in the first trimester;
(6) No other causes identified of nausea and vomiting, such as gastrointestinal or urinary tract, infections, etc.

### 2.5. Assessment of Other Variables

Information on covariables included socio-demographic characteristics (age, educational level, employment status, annual household income, smoking, alcohol drinking, and use of nutritional supplements) and pregnancy-related information (gestational weeks and parity). Physical activity was assessed by the International Physical Activity Questionnaire (IPAQ), a seven-item questionnaire, to calculate the average daily metabolic equivalent (MET). Pre-pregnant weight and height were measured using a self-administered questionnaire. Weight (kg) was divided by height squared (m^2^) to calculate body mass index (BMI). Given that our study population was Chinese pregnant women, we defined a BMI ≥ 24 kg/m^2^ as overweight by referring to Chinese health industry standards [27] and previous studies on the weight of Chinese pregnant women [28,29].

### 2.6. Statistical Analysis

Participants’ characteristics are presented as mean ± standard deviation or median/interquartile range for continuous variables, and frequency (percent) for categorical variables. A binary logistic regression analysis was performed to estimate odds ratios (ORs) and 95% confidence intervals (CIs) for the associations of DII scores (as tertiles and per SD increase) with HG. Three models were fit. No variables were adjusted in Model 1; Model 2 was adjusted for age (continuous), gestational weeks (continuous), parity (primigravida/multipara), and total energy intake (≤1171.2 kcal/d/1171.3–1683.5 kcal/d/≥1683.6 kcal/d); and Model 3 was further adjusted for pre-pregnancy BMI (<24 kg/m^2^/≥24 kg/m^2^), annual household income (<CNY 100,000/≥CNY 100,000), educational level (under college/college and higher), physical activity (<876/≥876 for median MET-min/week), employment status (no/yes), smoking (no/yes), alcohol drinking (no/yes), and use of nutritional supplements (no/yes). The variance inflation factor (VIF) was used to assess multicollinearity between the variables included in Model 3. The maximum VIF value was 1.4, which is an indication that multicollinearity is acceptable (all values < 10). The DII scores were also used as continuous variables in the logistic regression to obtain *p*-values for trends. A restricted cubic spline regression with three knots (25, 50, and 75%) was used for dose–response relationships between DII and HG. We performed stratified analyses defined by key demographic characteristics to test the heterogeneity of HG risk in different subgroups. Additionally, the interactions between these factors and the DII were assessed by adding their interaction terms to the final model. Moreover, to identify the primary contributors to the association between the DII and risk of HG, each food parameter of the DII was analyzed in relation to the outcome.

All statistical analyses were performed using SPSS (version 25; IBM Corp, Armonk, NY, USA). All the tests were two-sided, and *p* < 0.05 was considered statistically significant.

## 3. Results

**Figure A1:**
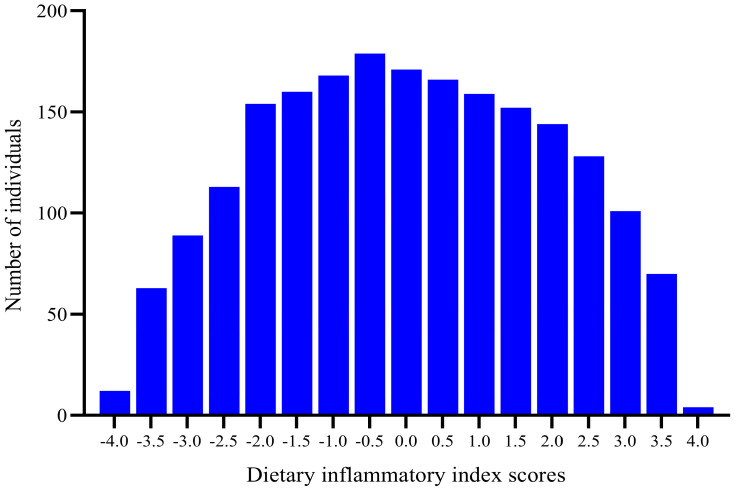
Distribution of the dietary inflammatory index among the study participants (*n* = 2033).

In this study, the DII scores ranged from −4.04 to +3.82, with the negative score indicating an anti-inflammatory diet, whereas a positive score indicates a pro-inflammatory diet. The median DII scores for the tertiles were −2.06, +0.01, and +2.11. Table 1 shows the distribution of the characteristics of the participants across the tertiles of the DII score. And Figure A1 shows the distribution of the DII in the study population. Significant differences were observed across the DII tertiles in terms of parity, total energy intake, physical activity, and use of nutrient supplements. The proportion of primiparous women increased progressively from 67.4% in the T1 to 74.8% in the T3. Participants in the T1 reported the highest total energy intake. The T2 group contained the lowest proportion of pregnant women with less physical activity. Additionally, the highest usage of nutrient supplements was noted in the T1. No significant differences were observed between the tertiles for age, weeks of pregnancy, pre-pregnancy BMI, annual household income, education level, employment status, smoking, or alcohol drinking. And, in this study, 8.2% (*n* = 167) of the pregnant women were diagnosed with HG. Table A1 presents the general characteristics of participants with and without HG. Apart from a significantly higher gestational age in the HG group, no other significant differences were found between the two groups in the analyzed variables.

Table A2 shows the dietary intake of participants across tertiles of DII score. The higher DII scores were significantly associated with lower intakes of energy, carbohydrate, total fat, protein, cholesterol, vitamin B12, iron, alcohol, MUFA, PUFA, fiber, vitamin B6, folic acid, niacin, riboflavin, thiamine, vitamin A, vitamin C, vitamin D, vitamin E, zinc, selenium, and magnesium.

We found that gestational weeks and total energy intake were positively associated with the odds of HG. The risk of HG increased by 19% with advancing gestational weeks. Compared to women with a total energy intake of 1171.2 kcal/d or less, those consuming between 1171.3 and 1683.5 kcal/d had a 1.74-fold increased risk of HG (95% CI: 1.12, 2.71). When the total energy intake exceeded 1683.6 kcal/d, the risk escalated to 2.35 (95% CI: 1.46, 3.77), as shown in Table 2.

**Figure 1:**
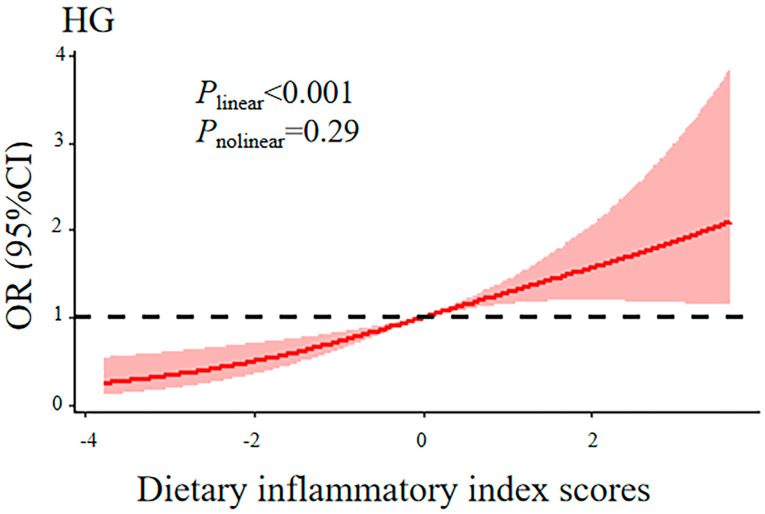
The restricted cubic spline for the association of dietary inflammatory index with hyperemesis gravidarum. Knots were placed at the 25th, 50th, and 75th percentiles of the dietary inflammatory index distribution. The solid red line represents the point estimate of the OR, and the red area represents the 95% confidence interval. Results were adjusted for age, gestational weeks, parity, total energy intake, physical activity, pre-pregnancy body mass index, annual household income, educational level, employment status, smoking, alcohol drinking, and use of nutritional supplements. OR: odds ratio, CI: confidence interval, HG: hyperemesis gravidarum.

The ORs and 95% CIs for the association between the DII scores and HG are provided in Table 3. In the initial crude model, no significant positive correlation was observed between DII scores, treated as a continuous variable, and the risk of HG (OR: 1.01; 95% CI: 0.86–1.18). However, after adjustments were made for age, gestational weeks, parity, and total energy intake, this correlation became significant (OR: 1.22; 95% CI: 1.01–1.48). All participants were divided into tertiles according to the levels of DII, and we found that this positive correlation (OR: 1.59; 95% CI: 1.01–2.51; *P*_trend_ = 0.046) was maintained. After adjusting for all covariates, the positive association between DII scores and HG was further strengthened (OR: 1.24; 95% CI: 1.03–1.50); in particular, the highest DII tertile had a 1.65-fold greater risk of HG than the lowest (95% CI: 1.04–2.62; *P*_trend_ = 0.032). Moreover, multivariable-adjusted restricted cubic spline regression indicated a linear increase in HG risk with increasing DII scores (*p* for non-linearity = 0.65) (Figure 1).

Table A3 shows the association between the remaining DII with 22 food components and the risk of HG after excluding individual food parameters. When PUFA (OR: 1.20; 95%CI: 0.99, 1.44), vitamin B6 (OR: 1.20; 95%CI: 1.00, 1.44), niacin (OR: 1.19; 95%CI: 0.99, 1.43), vitamin C (OR: 1.21; 95%CI: 1.00, 1.46), vitamin D (OR: 1.17; 95%CI: 0.97, 1.41), vitamin E (OR: 1.20; 95%CI: 1.00, 1.44), zinc (OR: 1.19; 95%CI: 0.99, 1.43), and selenium (OR: 1.20; 95%CI: 1.00, 1.44) were excluded, the association between DII and HG risk was not significant. In contrast, the association did not appreciably change when the other individual food parameters of the DII were excluded.

**Figure 2:**
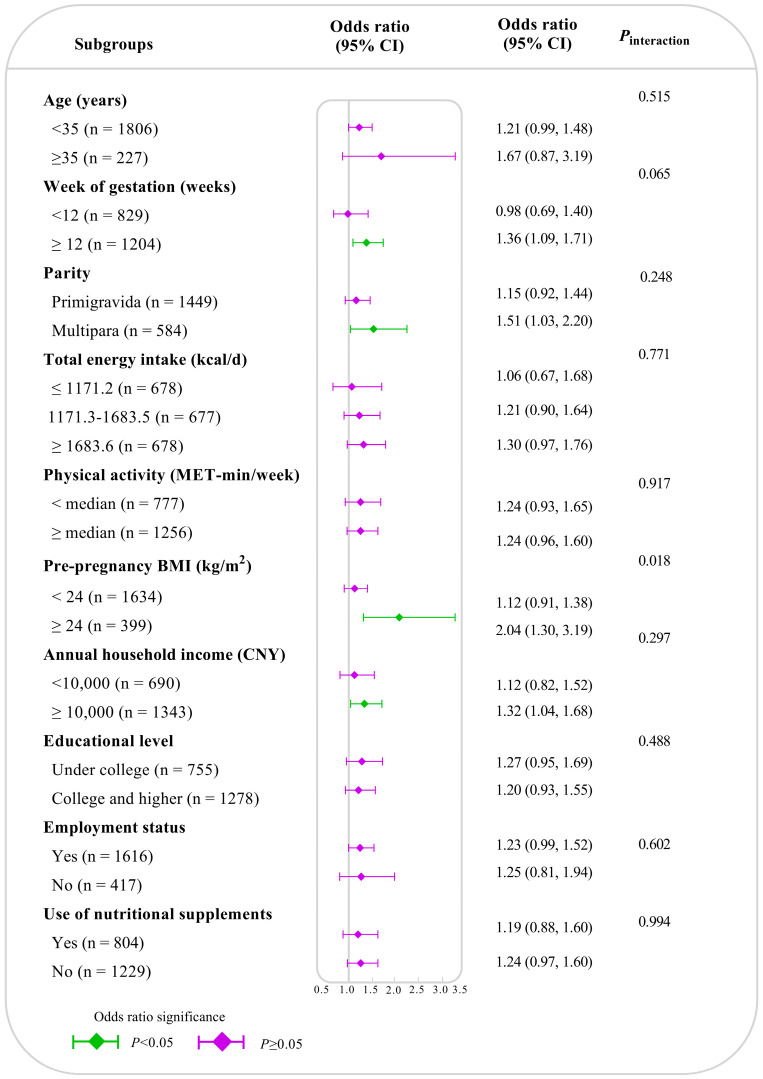
Subgroup analyses for the association of dietary inflammatory index with the risk of hyperemesis gravidarum. The association of dietary inflammatory index with hyperemesis gravidarum was linear, and thus we modeled DII as a continuous variable (per standard deviation increase) in the subgroup analysis. The left side shows the variables and their grouping, the gray vertical line indicates that the OR is 1, the diamond indicates the point estimate of the OR, the horizontal line represents the corresponding 95% CI, purple indicates no statistically significant difference, and green indicates that the difference is significant. We did not conduct a subgroup analysis of smoking and alcohol drinking due to the small number of smokers and alcohol drinkers. Multivariable binary logistic analyses were conducted by adjusting for age, gestational weeks, parity, total energy intake, physical activity, pre-pregnancy body mass index, annual household income, educational level, employment status, smoking, alcohol drinking, and use of nutritional supplements. OR: odds ratio, CI: confidence interval.

In the subgroup analyses (Figure 2), the positive association between the DII and risk of HG was stronger in those with pre-pregnancy overweight/obesity (OR: 2.04; 95% CI: 1.30–3.19; *P*_interaction_ = 0.018). In addition, such an association did not vary significantly in subgroups defined by age, week of gestation, parity, total energy intake, physical activity, annual household income, educational level, employment status, or the use of nutritional supplements (*P*_interaction_ ≥ 0.05).

## 4. Discussion

In this cross-sectional study, we found that after adjusting for potential confounders, higher DII scores indicating a proinflammatory diet were significantly associated with increased risk of HG. The association is stronger in pregnant women with pre-pregnancy overweight. Moreover, we identified eight dietary parameters that modulate the relationship between the DII and the risk of HG, providing potential mechanisms for the positive correlation between the DII and HG risk.

The range of DII scores for the present study (−4.04 to +3.82) was moderate compared to pregnant women around the world, higher than pregnant women in Iran (−0.50 to +2.70) [30] and Spain (−5.71 to −0.33) [31] while lower than pregnant women in Japan (−6.16 to +5.80) [32]. Compared with the study of pregnant women in China, the range of DII scores in our study was slightly lower than those reported by Yang et al. (−1.36 to +5.35) [14] and Zhang et al. (−5.35 to −6.77) [33]. In other words, the dietary anti-inflammatory capacity exhibited by the participants in this study is superior. This might be attributed to the fact that their studies encompassed the entire pregnancy period, while ours specifically concentrated on the early pregnancy stage. Moreover, the nutritional components included in the DII calculation of their studies were not entirely aligned with ours. Additionally, the DII in Zhang et al.’s study was referred to as energy-adjusted DII, and its calculation methodology was not entirely the same as that adopted in this study. Nevertheless, the range of DII scores observed in our study was comparable to those reported by another study of pregnant women in China (−4.45 to +3.15) [34].

We found a positive correlation between gestational weeks, high energy intake, and HG, which may be associated with the gradual increased human chorionic gonadotropin (hCG) and estradiol levels in pregnant women during the early stages of pregnancy. Studies found that hCG may stimulate secretion in the gastrointestinal tract (GIT), leading to excessive fluid secretion and accumulation, which in turn causes intestinal distension. The distension of GIT triggers the vomit reflex via mechanoreceptors [35,36]. In addition, high levels of oestradiol have been demonstrated to result in a reduction in both intestinal transit time and gastric emptying, which may contribute to the accumulation of fluid within the body [37]. This, in turn, alters the concentration of gastric acid, potentially increasing the risk of *Helicobacter pylori* (*Hp*) infection, which has been proven to be one of the pathogenic mechanisms of HG [35,38]. Furthermore, it must be noted that as it was a cross-sectional study, reverse causality could not be ruled out. In other words, patients with HG may increase their energy intake to alleviate prolonged nausea and vomiting [39]. Previous studies have found that compared to normal pregnant women, HG patients exhibit significantly higher ghrelin levels [40], which physiologically contributes to an increase in food intake [41].

To our knowledge, this represents the first research endeavor to explore the link between DII and the risk of HG. Recently, there has been heightened interest in exploring the significance of dietary inflammatory potential and its connection to pregnancy complications (e.g., GDM, miscarriage, preeclampsia, gestational hypertension, and preterm birth) [15,30,34]. However, no studies have explored the relationship with HG. Our study provides new evidence linking a pro-inflammatory diet to HG risk. Our findings were supported by several previous studies. Such studies have reported that increased consumption of pro-inflammatory components, such as total fat, elevates circulating estrogen levels, contributing to the incidence of HG [42]. In contrast, adherence to an anti-inflammatory diet rich in vegetables and fish has been associated with a lower risk of severe vomiting [43]. Additionally, it was demonstrated that women suffering from NVP exhibit significantly higher consumption of sugar-sweetened beverages [39], which is corroborated by a prospective cohort study from the UK, in which a positive association was found between the severity of nausea and increased consumption of carbonated drinks [44]. In consideration of the available evidence, it appears that a diet that promotes inflammation could be correlated with the emergence of HG. Nevertheless, the connection between the DII and HG has not been explored in any studies so far [45]. As one of the principal causes of hospitalization amongst pregnant women in the early stages of pregnancy, further investigation is required to ascertain whether a pro-inflammatory diet may elevate the development of HG.

In this study, we employed a 108-item FFQ to assess the dietary intake of pregnant women and included 23 food parameters to calculate the DII scores. Our findings indicate that a high DII score is associated with an increased risk of HG. Interestingly, our findings indicate that the positive association between the DII and the risk of developing HG is more pronounced among pregnant women who were overweight or obese before pregnancy. Obesity is known to correlate with long-term adherence to a pro-inflammatory diet [46]. Consequently, pre-pregnancy overweight may amplify the link between a pro-inflammatory diet and HG [47]. Further investigation of the relationship between a pro-inflammatory diet and the occurrence of HG over a range of pre-pregnancy body mass indexes is warranted.

After excluding specific components such as PUFA, vitamin B6, niacin, vitamin C, vitamin D, vitamin E, zinc, and selenium, the association between the DII and HG became insignificant, suggesting that these components contribute significantly to this association. PUFA has been shown to inhibit the development of HG by suppressing the expression of TNF-α and IL-6 through the inhibition of the nuclear factor-kappaB (NF-kB) signaling pathway [48]. Additionally, PUFAs might reduce the risk of HG by decreasing serum malondialdehyde levels, a classic marker of oxidative stress [49,50]. Vitamin B6 participates in over 160 enzymatic activities in the human body, including the metabolism of carbohydrates, glycogen, amino acids, unsaturated fatty acids, and nucleic acids [51]. It facilitates lysine reactivity (lysine being a protein residue of steroid hormone receptors), which mitigates nausea and vomiting caused by elevated estrogen levels in pregnant women. Thus, vitamin B6 is considered to be a beneficial supplement for malnourished patients and is also employed in the treatment of HG [8]. Niacin, another member of the B vitamin group, is sometimes co-administered with vitamin B6 for therapeutic purposes [52]. Research has also confirmed the association between vitamin D and HG [53]. A Mendelian randomization study demonstrated a causal relationship between increased levels of 25-hydroxyvitamin D and reduced risk of HG in European populations [54]. Vitamin D might suppress HG by inhibiting inflammatory responses during pregnancy. Vitamin C and E both act as antioxidants [55]. Zinc is essential for the activity of approximately 100 enzymes involved in various biological processes, which could reduce oxidative stress by maintaining the activity of antioxidant enzymes such as superoxide dismutase. Studies have also shown that people with HG have reduced zinc levels [56]. Selenium is an essential component of multiple antioxidant enzymes, such as glutathione peroxidase, which play a crucial role in scavenging free radicals and reducing oxidative stress [57]. These antioxidants may provide protective effects against HG by mitigating oxidative stress.

However, the specific pathways by which a diet promoting inflammation could impact the development of HG remain limited. While previous studies have mainly illustrated the mechanism in terms of a pro-inflammatory diet causing acid reflux and dyspepsia in pregnant women [9], it is also known that a pro-inflammatory diet is associated with a high level of inflammation in circulation. It has been proposed that inflammatory biomarkers such as TNF-α may bind to tumor necrosis factor-alpha receptor (TNF-α-R) and activate intracellular signal transduction mechanisms. TNF-α stimulates the IL-6-mediated signaling pathway by activating interleukin-6 receptor (IL-6-R), altering trophoblastic growth and function in early pregnancy [58], further stimulating the release of hCG, resulting in a significant increase in hCG during the 7th to 9th weeks of gestation [59,60], thereby inducing HG [35,61]. Additionally, IL-6, as a growth factor for trophoblastic cells [62], signals through the receptor subunit glycoprotein (GP) 130 and the downstream signaling molecule activator of transcription (STAT) 3 [63,64], inducing hCG to stimulate the corpus luteum to produce progesterone during pregnancy [65,66], thus contributing to the incidence of HG. Simultaneously, IL-1β stimulates prostaglandin E2 (PGE2) production from human placental tissue in a dose-dependent manner, causing nausea or vomiting [35,67]. High-sensitive C-reactive protein (hs-CRP), a sensitive marker of inflammation in the system, increases the production of reactive oxygen species (ROS), aggravating oxidative stress in the body [68,69], thus increasing the likelihood of HG. Therefore, cytokines may have a potential role to play in the pathology of HG caused by inflammation [70].

This study possesses several strengths, particularly in being the first to explore the association between DII scores and the HG risk. In addition, we included a large number of pregnant women from northwestern China, whose dietary characteristics can represent the dietary habits of pregnant women in western China [20]. However, this study also has some limitations. Firstly, since food ingredients such as eugenol, saffron, and rosemary are not commonly consumed in Chinese residents’ diets and there is no relevant information in the Chinese Food Composition Database [34], and the FFQ used in this study lacks information on the consumption of cooking ingredients such as pepper, trans fat, and turmeric [33], only 23 food items were ultimately used for DII calculation in this study. However, previous studies have verified that utilizing fewer than 30 food components to calculate the DII score does not diminish its predictive capability [33,71]. Secondly, the DII score was calculated from the FFQ, which may result in misclassification of dietary intake. However, studies have shown that the FFQ can be used to assess dietary intake during early pregnancy, and dietary intake at a certain point during pregnancy can reflect dietary characteristics throughout pregnancy [9,72]. Thirdly, we did not use inflammatory biomarkers to validate the DII, and thus future studies need to further verify the reliability of the results by combining inflammatory biomarkers. Fourthly, the cross-sectional design inherently limits the study’s capacity to fully exclude residual confounding and precludes definitive causal inferences. Therefore, prospective studies are imperative for confirming our findings. Finally, the study participants consisted of Chinese pregnant women, so the findings might not be extrapolated to other populations.

## 5. Conclusions

To conclude, our study reveals that Chinese pregnant women with higher DII scores are at a greater risk of developing HG. This finding suggests that avoiding pro-inflammatory diets may reduce the occurrence of HG. Our study added epidemiological evidence for developing relevant prevention strategies and measures against HG. Nevertheless, there is a need for further prospective studies for confirmation of our findings.

## Figures and Tables

**Table 1:** General characteristics of participants across tertiles of the dietary inflammatory index scores.

Characteristic	Tertiles of Dietary Inflammatory Index Scores			*p*
	T1 (*n* = 678)	T2 (*n* = 677)	T3 (*n* = 678)	
Age (years)	31.4 ± 3.5	31.3 ± 3.4	31.1 ± 3.4	0.160
Week of gestation (weeks), median (IQR)	12.2 (10.0, 12.7)	12.2 (9.7, 12.7)	12.0 (9.5, 12.7)	0.533
Parity (primigravida), *n* (%)	457 (67.4)	485 (71.6)	507 (74.8)	0.011
Total energy intake (kcal/d), median (IQR)	2036.6 (1713.3, 2510.2)	1403.7 (1214.3, 1633.1)	944.9 (772.3, 1114.0)	<0.001
Physical activity (<median), *n* (%)	274 (40.4)	231 (34.1)	272 (40.1)	0.027
Pre-pregnancy BMI (<24 kg/m^2^), *n* (%)	549 (81.0)	533 (78.7)	552 (81.4)	0.410
Annual household income (<CNY 100,000), *n* (%)	216 (31.9)	229 (33.8)	245 (36.1)	0.250
Educational level (under college), *n* (%)	255 (37.6)	236 (34.9)	264 (38.9)	0.285
Employment status (no), *n* (%)	143 (21.1)	137 (20.2)	137 (20.2)	0.900
Smoking (yes), *n* (%)	20 (2.9)	20 (3.0)	21 (3.1)	0.984
Alcohol drinking (yes), *n* (%)	21 (3.1)	22 (3.2)	27 (4.0)	0.634
Use of nutritional supplements (yes), *n* (%)	298 (44.0)	255 (37.7)	251 (37.0)	0.016
DII, median (IQR)	−2.06 (−2.74, −1.55)	+0.01 (−0.52, +0.50)	+2.11 (+1.55, +2.76)	

Note. *p*-values were derived from a one-way ANOVA test or Wilcoxon’s rank sum test (continuous variables) or Chi-squared test (categorical variables). IQR: interquartile range, BMI: body mass index, DII: dietary inflammatory index.

**Table 2:** Association between covariates in the model with hyperemesis gravidarum.

Variables	Number	HG	*p*
Age (continuous)	2033	1.02 (0.96, 1.07)	0.592
Week of gestation (continuous)	2033	1.19 (1.10, 2.10)	<0.001
Parity ^a^ (primigravida/multipara)	1449/584	0.97 (0.65, 1.44)	0.864
Total energy intake	2033		
≤1171.2 kcal/d	678	1.00	
1171.3–1683.5 kcal/d	677	1.74 (1.12, 2.71)	0.014
≥1683.6 kcal/d	678	2.35 (1.46, 3.77)	<0.001
Physical activity ^b^ (<median/≥median)	777/1256	0.99 (0.71, 1.38)	0.951
Pre-pregnancy BMI ^c^ (<24 kg/m^2^/≥24 kg/m^2^)	1634/399	0.98 (0.65, 1.47)	0.913
Annual household income ^d^ (<CNY 100,000/≥CNY 100,000)	690/1343	0.88 (0.62, 1.25)	0.467
Educational level ^e^ (under college/college and higher)	755/1278	0.74 (0.52, 1.06)	0.103
Employment status ^f^ (no/yes)	417/1616	1.04 (0.68, 1.58)	0.872
Smoking ^f^ (no/yes)	1972/61	0.53 (0.16, 1.76)	0.299
Alcohol drinking ^f^ (no/yes)	1963/70	0.66 (0.23, 1.87)	0.431
Use of nutritional supplements ^f^ (no/yes)	1229/804	1.30 (0.94, 1.80)	0.120

Note. HG: hyperemesis gravidarum. ^a^ Reference group: primigravida. ^b^ Reference group: <median. ^c^ Reference group: <24 kg/m^2^. ^d^ Reference group: <CNY 100,000. ^e^ Reference group: under college. ^f^ Reference group: no.

**Table 3:** Associations between the dietary inflammatory index and hyperemesis gravidarum risk.

Model	Tertiles of Dietary Inflammatory Index Scores (OR, 95%CI)			*P*_trend_	Per SD Increase
	T1 (≤−1.00)	T2 (−0.99~0.97)	T3 (≥0.98)		
case/control	53/625	60/617	54/624		
Model 1	1.00 (Ref.)	1.15 (0.78, 1.69)	1.02 (0.69, 1.52)	0.921	1.01 (0.86, 1.18)
Model 2	1.00 (Ref.)	1.42 (0.95, 2.12)	1.59 (1.01, 2.51)	0.046	1.22 (1.01, 1.48)
Model 3	1.00 (Ref.)	1.48 (0.98, 2.22)	1.65 (1.04, 2.62)	0.032	1.24 (1.03, 1.50)

Note. Model 1: crude, no adjustment; Model 2: adjusting for age, gestational weeks, parity, and total energy intake; Model 3: adjusting for age, gestational weeks, parity, total energy intake, physical activity, pre-pregnancy body mass index, annual household income, educational level, employment status, smoking, alcohol drinking, and use of nutritional supplements. OR: odds ratio, CI: confidence interval, SD: standard deviation.

## Data Availability

Due to data protection restrictions, the data are only available on request from the corresponding author of the data presented in this study.

## Appendix A

**Table A1:** General characteristics of HG and non-HG groups.

Characteristic	Participants	Non-HG (*n* = 1866)	HG (*n* = 167)	*p*
Age (years)	31.3 ± 3.4	31.3 ± 3.4	31.2 ± 3.5	0.856
Week of gestation (weeks), median (IQR)	12.0 (9.7, 12.7)	12.0 (9.3, 12.7)	12.3 (11.5, 12.7)	<0.001
Parity (primigravida), *n* (%)	1449 (71.3)	1330 (71.3)	119 (71.3)	0.996
Total energy intake (kcal/d), median (IQR)	1413.0 (1049.3, 1872.2)	1407.9 (1050.1, 1875.2)	1421.9 (1024.8, 1816.5)	0.911
Physical activity (<median MET-min/week), *n* (%)	777 (38.2)	713 (38.2)	64 (38.3)	0.977
Pre-pregnancy BMI (<24 kg/m^2^), *n* (%)	1634 (80.4)	1500 (80.4)	134 (80.2)	0.964
Annual household income (<CNY 100,000), *n* (%)	690 (33.9)	627 (33.6)	63 (37.7)	0.281
Educational level (under college), *n* (%)	755 (37.1)	683 (36.6)	72 (43.1)	0.095
Employment status (no), *n* (%)	417 (20.5)	381 (20.4)	36 (21.6)	0.727
Smoking (yes), *n* (%)	61 (3.0)	58 (3.1)	3 (1.8)	0.341
Alcohol drinking (yes), *n* (%)	70 (3.4)	66 (3.5)	4 (2.4)	0.438
Use of nutritional supplements (yes), *n* (%)	804 (39.5)	729 (39.1)	75 (44.9)	0.139
DII, median (IQR)	+0.01 (−1.55, +1.56)	−0.02 (−1.56, +1.56)	+0.10 (−1.46, +1.41)	0.890

Note. *p*-values were derived from an independent-sample *t*-test or Wilcoxon’s rank sum test (continuous variables) or Chi-squared test (categorical variables). HG: hyperemesis gravidarum, MET: metabolic equivalent, BMI: body mass index, IQR: interquartile range, DII: dietary inflammatory index.

**Table A2:** Dietary intakes of study participants across tertiles of dietary inflammatory index.

Variables	Tertiles of Dietary Inflammatory Index Scores			*p*
	T1 (*n* = 678)	T2 (*n* = 677)	T3 (*n* = 678)	
Carbohydrate (g/d)	277.80 (225.12, 350.47)	195.99 (167.05, 236.79)	136.19 (110.8, 167.06)	<0.001
Total fat (g/d)	62.96 (49.93, 81.34)	41.79 (33.47, 51.71)	26.22 (20.34, 33.60)	<0.001
Protein (g/d)	102.38 (83.84, 127.36)	64.02 (56.31, 74.74)	41.08 (32.57, 48.81)	<0.001
Cholesterol (mg/d)	389.33 (264.91, 489.69)	262.89 (180.24, 375.13)	167.37 (86.39, 241.47)	<0.001
Vitamin B12 (μg/d)	0.71 (0.48, 1.05)	0.53 (0.41, 0.80)	0.44 (0.32, 0.64)	<0.001
Fe (mg/d)	24.50 (20.26, 30.67)	14.91 (13.13, 17.12)	9.45 (7.79, 10.96)	<0.001
Alcohol (g/d)	0.68 (0.68, 0.68)	0.68 (0.68, 0.68)	0.68 (0.68, 0.68)	0.038
MUFA (g/d)	15.88 (11.87, 22.22)	10.36 (7.31, 13.44)	5.61 (4.08, 8.65)	<0.001
PUFA (g/d)	9.22 (6.79, 11.98)	4.94 (3.93, 6.44)	2.87 (2.22, 3.68)	<0.001
Fiber (g/d)	16.53 (13.51, 21.33)	9.50 (8.10, 11.35)	5.34 (4.25, 6.55)	<0.001
Vitamin B6 (mg/d)	1.84 (1.51, 2.46)	1.17 (0.97, 1.36)	0.69 (0.57, 0.88)	<0.001
Folic acid (mg/d)	551.21 (467.28, 701.22)	331.34 (288.62, 377.46)	194.74 (155.40, 233.71)	<0.001
Niacin (mg/d)	19.55 (15.67, 26.07)	12.72 (10.56, 16.07)	8.340 (6.89, 10.29)	<0.001
Riboflavin (mg/d)	1.30 (1.07, 1.65)	0.93 (0.72, 0.97)	0.51 (0.40, 0.63)	<0.001
Thiamin (mg/d)	1.05 (0.88, 1.32)	0.67 (0.57, 0.75)	0.40 (0.31, 0.48)	<0.001
Vitamin A (RE)	902.59 (651.66, 1182.75)	485.94 (364.11, 643.09)	270.08 (213.20, 355.35)	<0.001
Vitamin C (mg/d)	224.00 (174.29, 296.98)	133.50 (103.76, 166.63)	73.33 (53.81, 98.46)	<0.001
Vitamin D (μg/d)	2.07 (1.06, 2.70)	1.22 (0.48, 2.21)	0.52 (0.289, 1.24)	<0.001
Vitamin E (mg/d)	33.17 (23.11, 42.39)	16.56 (13.76, 19.46)	9.65 (7.94, 12.29)	<0.001
Zn (mg/d)	12.66 (10.55, 16.01)	8.00 (6.92, 9.46)	5.14 (4.14, 6.10)	<0.001
Se (mg/d)	45.73 (36.64, 62.33)	30.94 (25.81, 38.23)	20.27 (16.25, 25.19)	<0.001
Mg (mg/d)	504.54 (433.42, 633.17)	318.90 (285.40, 350.73)	200.38 (165.93, 230.61)	<0.001

Note. Data are median and interquartile range [M (P75–P25)]; *p*-value was derived from Wilcoxon’s rank sum test; MUFA: monounsaturated fatty acids; PUFA: polyunsaturated fatty acids.

**Table A3:** Adjusted associations between dietary inflammatory index scores (excluding each component individually) and the risk of hyperemesis gravidarum.

Model	Tertiles of Dietary Inflammatory Index Scores (OR, 95%CI)			*P*_trend_	Per SD Increase
	T1	T2	T3		
DII-23	1.00 (Ref.)	1.48 (0.98, 2.22)	1.65 (1.04, 2.62)	0.032	1.24 (1.03, 1.50)
DII-except Energy	1.00 (Ref.)	1.43 (0.95, 2.14)	1.64 (1.03, 2.62)	0.035	1.23 (1.02, 1.48)
DII-except Carbohydrate	1.00 (Ref.)	1.42 (0.94, 2.12)	1.62 (1.02, 2.59)	0.038	1.22 (1.01, 1.47)
DII-except Total fat	1.00 (Ref.)	1.41 (0.94, 2.12)	1.62 (1.02, 2.58)	0.040	1.22 (1.01, 1.47)
DII-except Protein	1.00 (Ref.)	1.42 (0.94, 2.12)	1.61 (1.01, 2.56)	0.041	1.22 (1.01, 1.47)
DII-except Cholesterol	1.00 (Ref.)	1.41 (0.94, 2.12)	1.61 (1.01, 2.55)	0.042	1.21 (1.01, 1.46)
DII-except Vitamin B12	1.00 (Ref.)	1.40 (0.93, 2.10)	1.67 (1.05, 2.66)	0.029	1.23 (1.02, 1.49)
DII-except Fe	1.00 (Ref.)	1.47 (0.98, 2.21)	1.65 (1.03, 2.62)	0.032	1.23 (1.02, 1.48)
DII-except Alcohol	1.00 (Ref.)	1.37 (0.91, 2.05)	1.69 (1.07, 2.66)	0.025	1.24 (1.03, 1.49)
DII-except MUFA	1.00 (Ref.)	1.48 (0.98, 2.22)	1.64 (1.03, 2.61)	0.033	1.23 (1.02, 1.48)
DII-except PUFA	1.00 (Ref.)	1.37 (0.91, 2.05)	1.54 (0.98, 2.44)	0.060	1.20 (0.99, 1.44)
DII-except Fiber	1.00 (Ref.)	1.49 (0.99, 2.24)	1.64 (1.03, 2.61)	0.033	1.23 (1.02, 1.48)
DII-except Vitamin B6	1.00 (Ref.)	1.50 (1.00, 2.24)	1.55 (0.98, 2.46)	0.054	1.20 (1.00, 1.44)
DII-except Folic acid	1.00 (Ref.)	1.39 (0.92, 2.08)	1.65 (1.04, 2.62)	0.031	1.23 (1.02, 1.48)
DII-except Niacin	1.00 (Ref.)	1.50 (1.00, 2.24)	1.54 (0.97, 2.43)	0.059	1.19 (0.99, 1.43)
DII-except Riboflavin	1.00 (Ref.)	1.44 (0.96, 2.16)	1.67 (1.06, 2.65)	0.027	1.24 (1.03, 1.49)
DII-except Thiamin	1.00 (Ref.)	1.44 (0.96, 2.17)	1.67 (1.06, 2.65)	0.027	1.23 (1.02, 1.49)
DII-except Vitamin A	1.00 (Ref.)	1.35 (0.89, 2.02)	1.72 (1.09, 2.72)	0.020	1.25 (1.04, 1.50)
DII-except Vitamin C	1.00 (Ref.)	1.33 (0.89, 1.99)	1.59 (1.00, 2.52)	0.047	1.21 (1.00, 1.46)
DII-except Vitamin D	1.00 (Ref.)	1.54 (1.03, 2.29)	1.46 (0.92, 2.32)	0.097	1.17 (0.97, 1.41)
DII-except Vitamin E	1.00 (Ref.)	1.37 (0.91, 2.06)	1.56 (0.98, 2.47)	0.056	1.20 (1.00, 1.44)
DII-except Zn	1.00 (Ref.)	1.44 (0.96, 2.15)	1.52 (0.96, 2.41)	0.068	1.19 (0.99, 1.43)
DII-except Se	1.00 (Ref.)	1.42 (0.95, 2.13)	1.56 (0.99, 2.46)	0.054	1.20 (1.00, 1.44)
DII-except Mg	1.00 (Ref.)	1.32 (0.88, 1.99)	1.61 (1.03, 2.54)	0.038	1.22 (1.01, 1.46)

Note. Model was adjusted for age, gestational weeks, total energy intake, parity, physical activity, pre-pregnancy body mass index, annual household income, employment status, educational level, smoking, alcohol drinking, and use of nutritional supplements. DII: dietary inflammatory index, OR: odds ratio, CI: confidence interval, SD: standard deviation.
